# Outcomes of leadless pacemaker implantation in the United States based on sex

**DOI:** 10.1007/s10840-024-01936-2

**Published:** 2024-10-22

**Authors:** Muhammad Zia Khan, Bandar Alyami, Waleed Alruwaili, Amanda T. Nguyen, Melody Mendez, William E. Leon, Justin Devera, Hafiz Muhammad Sohaib Hayat, Abdullah Naveed, Zain Ul Abideen Asad, Siddharth Agarwal, Sudarshan Balla, Douglas Darden, Muhammad Bilal Munir

**Affiliations:** 1 Division of Cardiology, West Virginia University Heart and Vascular Institute, Morgantown, WV USA; 2 Division of Cardiology, University of California Davis, Sacramento, CA USA; 3 Department of Medicine, Dow Medical College, Karachi, Sindh Pakistan; 4 Division of Cardiology, University of Oklahoma, Oklahoma City, OK USA; 5 Division of Cardiology, Kansas City Heart Rhythm Institute, Overland Park, KS USA; 6 Section of Electrophysiology, Division of Cardiology, University of California Davis, Sacramento, CA USA

**Keywords:** Leadless, Pacemaker, Women, Outcomes, Complications, Mortality

## Abstract

**Background:**

To determine differences in baseline characteristics and outcomes of leadless pacemaker implantation based on sex.

**Methods:**

For the purpose of this study, data were extracted from the National Inpatient Sample database for years 2016–2020. The study group was then stratified based on sex. Baseline characteristics and in-hospital outcomes including complications were then analyzed in each group. Multivariable logistic regression models were created to analyze the association of sex with important outcomes of mortality, major complications (defined as pericardial effusion requiring intervention and any vascular complication), prolonged length of stay (defined as > 6 days), and increased cost of hospitalization (defined as median cost > 34,098$) after leadless pacemaker implantation.

**Results:**

A total of 29,000 leadless pacemakers (*n* in women = 12,960, 44.7%) were implanted during our study period. Women were found to have an increased burden of co-morbidities as compared to men. In the adjusted analysis, the likelihood of mortality (aOR 1.27, 95% CI 1.14–1.43), major complications (aOR 1.07, 95% CI 0.98–1.18), prolonged length of stay (aOR 1.09, 95% CI 1.04–1.15), and increased hospitalization cost (aOR 1.14, 95% CI 1.08–1.20) were higher in women as compared to men after leadless pacemaker implantation.

**Conclusion:**

Important and significant differences exist in leadless pacemaker implantation in women as compared to men. These findings highlight the need for evaluating etiologies behind such differences with a goal of improving outcomes in all patients after leadless pacemaker implantation.

**Graphical Abstract:**

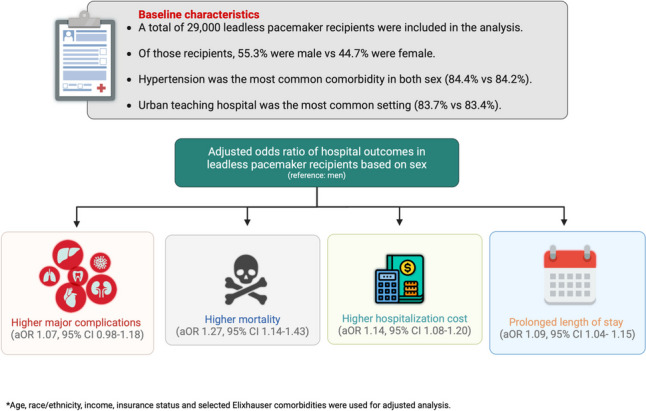

## Introduction

Leadless pacemakers (LPMs) represent a significant milestone in the management of patients with symptomatic bradyarrhythmia and are especially beneficial for individuals at risk of infection and with limited upper extremity vascular access [1, 2]. Earlier studies have shown significant sex-based disparities with respect to delivery of invasive cardiovascular care [3, 4]. Women were found to have a lower utilization of conventional transvenous pacemakers as compared to men when implanted for a similar indication and were also at an increased risk of acute complications from the procedure [5, 6]. There is, however, paucity of large-scale and contemporary studies on the utilization and safety of LPMs based on sex. In this study, we aimed to evaluate sex differences in the utilization and in-hospital outcomes of patients who underwent leadless pacemaker implantation from a large and nationally representative US population database.

## Methods

### Data source

For our study, we utilized data from the National Inpatient Sample (NIS) encompassing years 2016–2020. On April 6, 2016, the Food and Drug Administration (FDA) approved the first leadless pacemaker device, the Medtronic Micra [7]. The NIS is a large hospital based administrative database which was made possible by a Federal-State-Industry partnership sponsored by the Agency for Healthcare Research and Quality. The NIS can be used for computing national estimates of healthcare utilization, costs, and outcomes. The NIS provides discharge weights that are used for estimation of disease and procedure trends nationally. The data is de-identified; therefore, the need for informed consent and Institutional Review Board approval is waived [8]. The NIS adheres to the 2013 Declaration of Helsinki for the conduct of human research.

### Study population

We used the International Classification of Diseases, 10th Revision, Clinical Modification (ICD-10-CM) code 02HK3NZ, to identify patients who underwent leadless pacemaker implantation during our study period while excluding patients under 18 years of age and those lacking demographic information. The study cohort was then stratified by sex into men and women as defined in the database. We evaluated baseline characteristics, procedural complications, and other in-hospital outcomes such as mortality (treated as a separate category in the data), length of stay, and cost associated with hospitalization. We also evaluated the independent association of sex with significant outcomes of mortality, major complications (defined as a composite of pericardial effusion requiring intervention and vascular complications [AV fistula, pseudoaneurysm, access site hematoma, retroperitoneal bleeding, and venous thromboembolism]), prolonged length of stay (defined as > 6 days), and higher hospitalization costs (defined as median cost > 34,098$). For computing hospitalization costs, the cost-to-charge ratio files based on Center for Medicare and Medicaid Services reimbursement and provided by the Healthcare Cost and Utilization Project were applied to the total hospital charges.

### Statistical analysis

Descriptive statistics were analyzed using frequencies and percentages for categorical variables, and as median with interquartile range (IQR) for continuous data. Baseline characteristics were compared using a Pearson X2 test and Fisher exact test for categorical variables and the Kruskal–Wallis H test for continuous variables. For crude comparison of procedural complications and in-hospital outcomes among the study groups, the Pearson *χ*^2^ test was used.

For the assessment of independent association of sex with outcomes of mortality, major complications, prolonged length of stay, and increased hospitalization costs, a single-step multivariable logistic regression model was used. Age, race/ethnicity, income, insurance status, and selected Elixhauser comorbidities were used for adjusted analysis. All these covariates were identified based on prior literature, bivariate analysis, and authors’ best clinical judgement [9–11]. A *p*-value of < 0.05 was considered statistically significant. All statistical analyses were performed using SPSS version 26 (IBM Corp, Armonk, NY) and R version 3.6. Because of the complex survey design of the NIS, sample weights, strata, and clusters were applied to raw data to generate national estimates [8].

## Result

### Baseline characteristics

A total of 29,000 LPM implantations were included in the study. Out of these, approximately 16,040 (55.3%) implantations occurred in men and 12,960 (44.7%) implantations occurred in women. Baseline characteristics of the study population are shown in Table 1. Women undergoing LPM implantations were found to have a higher burden of important comorbidities when compared to men. The prevalence of heart failure (53.9% vs. 50.9%, *p* < 0.01), chronic pulmonary disease (26% vs. 23.1%, *p* < 0.01), and hypothyroidism (26.9% vs. 14.4%, *p* < 0.01) were higher in women as compared to men. Urban teaching was the most common hospital location for LPM implantations for both men (83.7%) and women (83.4%).

**Table 1:** Baseline characteristics of the study population

Variable no. (%)	Men 16,040 (55.3)	Women 12,960 (44.7)	
Age (median [IQR]) years	77 (68–84)	79 (70–86)	< 0.01
Age < 65	2960 (18.5)	1985 (15.3)	< 0.01
65–74	3750 (23.4)	2725 (21.0)	
≥ 75	9330 (58.2)	8250 (63.7)	
Race			
White	12,165 (78.0)	9400 (74.6)	< 0.01
Black	1430 (9.2)	1360 (10.8)	
Hispanic	1105 (7.1)	985 (7.8)	
Asian or Pacific Islander	440 (2.8)	450 (3.6)	
Native American	65 (0.4)	45 (0.4)	
Other	390 (2.5)	365 (2.9)	
Comorbidities			
Deficiency anemia	810 (5.0)	850 (6.6)	< 0.01
Congestive heart failure	8170 (50.9)	6980 (53.9)	< 0.01
Chronic pulmonary disease	3700 (23.1)	3365 (26.0)	< 0.01
Cerebrovascular disorders	1695 (10.60%	1395 (10.80%	0.52
Coagulopathy	2250 (14.0)	1540 (11.9)	< 0.01
Coronary artery disease	8145 (50.8)	4490 (34.6)	< 0.01
Diabetes mellitus	1540 (9.6)	1295 (10.0)	0.27
Drug abuse	400 (2.5)	280 (2.2)	0.06
Hypertension	13,530 (84.4)	10,910 (84.2)	0.69
Hypothyroidism	2315 (14.4)	3485 (26.9)	< 0.01
Liver disease	1010 (6.3)	790 (6.1)	0.48
Obesity	2870 (17.9)	2310 (17.8)	0.88
Peripheral vascular disorders	1720 (10.7)	1245 (9.6)	< 0.01
Chronic kidney disease	7055 (44.0)	4785 (36.9)	< 0.01
Pathological weight loss	1570 (9.8)	1455 (11.2)	< 0.01
Hospital location			
Rural	395 (2.5)	270 (2.1)	0.04
Urban non-teaching	2225 (13.9)	1880 (14.5)	
Urban teaching	13,420 (83.7)	10,810 (83.4)	
Bed size of the hospital			
Small	1605 (10.0)	1355 (10.5)	0.03
Medium	3785 (23.6)	3195 (24.7)	
Large	10,650 (66.4)	8410 (64.9)	
Payee			
Medicare	12,745 (79.6)	10,975 (84.7)	< 0.01
Medicaid	830 (5.2)	680 (5.3)	
Private insurance	1765 (11.0)	1170 (9.0)	
Self-pay	195 (1.2)	75 (0.6)	
No charge	20 (0.1)	10 (0.1)	
Other	455 (2.8)	40 (0.3)	
Median income			
0–25	3875 (24.5)	3440 (26.8)	< 0.01
25–50	4115 (26.1)	3375 (26.3)	
50–75	4005 (25.4)	3000 (23.4)	
75–100	3800 (24.1)	3000 (23.4)	

For *N* < 10, the absolute numbers are not reported as per Healthcare Cost and Utilization Project recommendations

### Procedure-related complications

The crude rate of procedure-related complications after LPM implantations and stratified on the basis of sex is shown in Table 2. The rate of pericardial effusion (4.7% vs. 2.3%, *p* < 0.01) and pericardial effusion requiring intervention (1.5% vs. 0.8%, *p* < 0.01) was higher in women as compared to men after LPM implantations. The rate of any peripheral vascular complication was similar in both women and men (7.4% vs. 7.8%, *p* = 0.15). The rate of acute kidney injury after LPM implantation was higher in men when compared to women (31.8% vs. 29.8%, *p* < 0.01).

**Table 2:** Leadless pacemaker procedure related complications stratified by sex

Variables no. (%)	Men 16,040 (55.3)	Women 12,960 (44.7)	*p*-value
Major complications*	1370 (8.5)	1120 (8.6)	0.76
Pericardial effusion requiring intervention	130 (0.8)	195 (1.5)	< 0.01
Pericardial effusion	370 (2.3)	615 (4.7)	< 0.01
Any peripheral vascular complication**	1255 (7.8)	955 (7.4)	0.15
AV fistula	35 (0.2)	40 (0.3)	0.13
Pseudoaneurysm	120 (0.7)	120 (0.9)	0.10
Hematoma	245 (1.5)	215 (1.7)	0.37
Retroperitoneal bleeding	70 (0.4)	35 (0.3)	0.02
Venous thromboembolism	850 (5.3)	575 (4.4)	< 0.01
Acute kidney injury	5100 (31.8)	3860 (29.8)	< 0.01

*Defined as a composite of pericardial effusion requiring intervention and vascular complications (AV fistula, pseudoaneurysm, access site hematoma, retroperitoneal bleeding, and venous thromboembolism)

**Defined as a composite of AV fistula, pseudoaneurysm, hematoma, retroperitoneal bleeding, and venous thromboembolism

### Hospital outcomes

The crude rate of other hospital-related outcomes after LPM implantations and stratified on the basis of sex is shown in Table 3. The crude rate of in-hospital mortality was higher in women as compared to men after LPM implantations (5.5% vs. 4.5%, *p* < 0.01). The rate of non-home discharges was also higher for women as compared to men after LPM implantations (35.9% vs. 31.8%, *p* < 0.01).

**Table 3:** Hospital outcomes in leadless pacemaker recipients stratified by sex

Variables no. (%)	Men 16,040 (55.3)	Women 12,960 (44.7)	*p*-value
Died at discharge	725 (4.5)	715 (5.5)	< 0.01
Discharge disposition			
Home/routine/self-care	10,450 (68.2)	7850 (64.1)	< 0.01
Nonhome discharges	4865 (31.8)	4395 (35.9)	
Resource utilization, median (IQR)			
Length of stay, days	6 (3–11)	6 (3–11)	< 0.01
Cost of hospitalization, $	34,067 (23,162–56,593)	34,118 (23,680–55,110)	< 0.01

For *N* < 10, the absolute numbers are not reported as per Healthcare Cost and Utilization Project recommendations

### Adjusted outcomes stratified on the basis of sex after LPM implantations

**Fig. 1:**
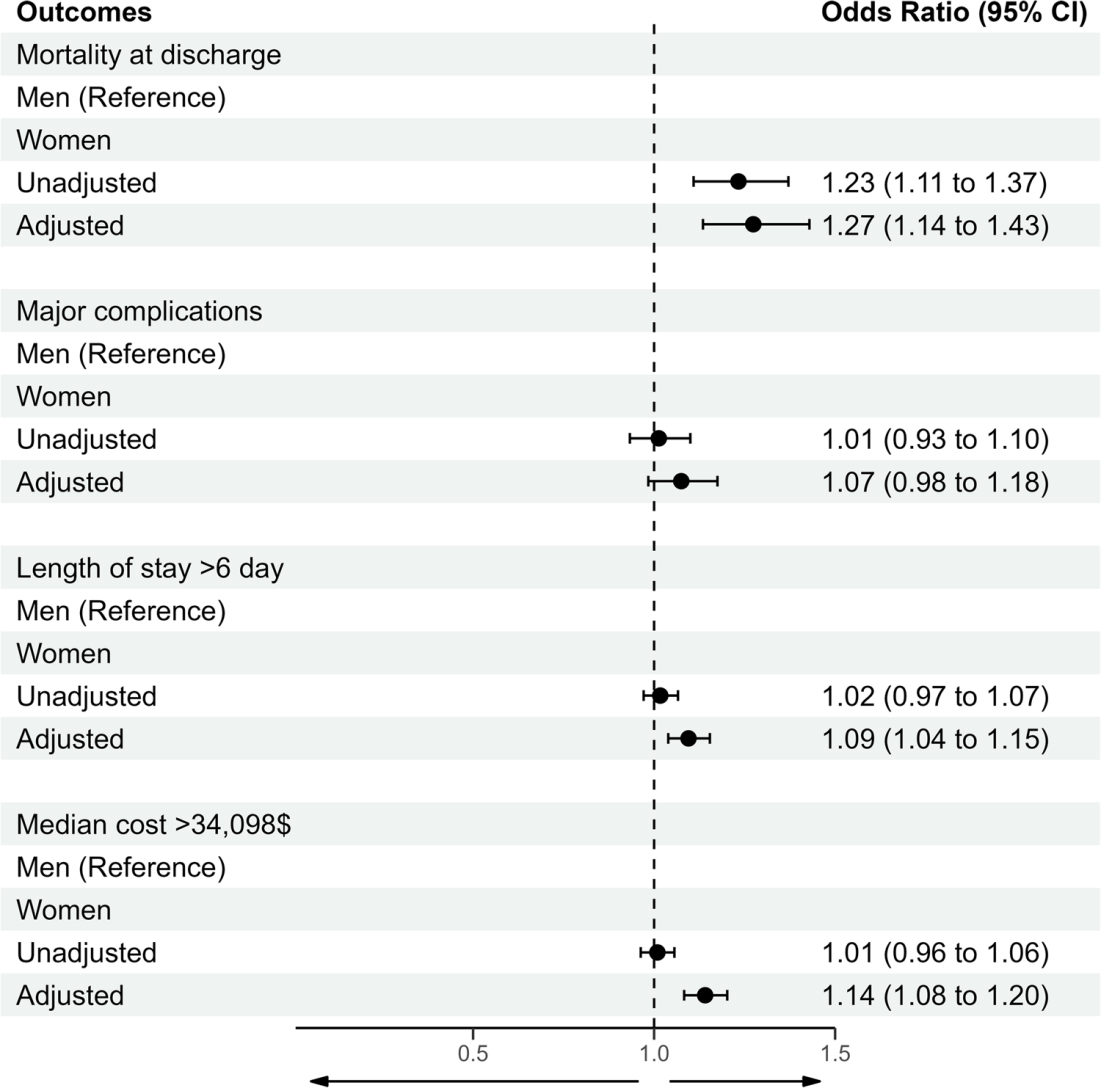
Adjusted association of sex with outcomes of mortality, major complications, prolonged length of stay, and increased hospitalization costs after leadless pacemaker implantation

To assess the independent association of sex with outcomes, we constructed multivariable models adjusting for potential confounders and are shown in Fig. 1. After adjustment, women were associated with higher in-hospital mortality (OR 1.27, 95% CI 1.14–1.43), major complications (OR 1.07, 95% CI 0.98–1.18), prolonged length of stay (OR 1.09, 95% CI 1.04–1.15), and increased cost of hospitalization (OR 1.14, 95% CI 1.08–1.20).

## Discussion

The main findings of our current investigation are as follows: (1) approximately 44.7% of women were implanted with a LPM which is lower when compared to men (55.3%). (2) Women undergoing LPM implantations were older and had a higher burden of important co-morbidities when compared to men. (3) Women were found to have an increased risk of mortality, major complications (statistically equivalent in adjusted analysis with crude analysis showing an increased risk of pericardial effusion), prolonged length of stay, and increased cost of hospitalization after LPM implantation when compared to men.

LPMs have emerged as an important therapeutic modality in management of patients with pacing indications and are especially useful in patients at risk of infection and with upper extremity vascular access issues. Prior literature has shown significant disparities with respect to delivery of invasive cardiovascular therapies for women as compared to men. In a study of more than 17,000 patients undergoing conventional transvenous pacemakers, Nowak et al. demonstrated that only 47% of women underwent such implantations when compared to men for similar underlying bradyarrhythmia indication [5]. The pivotal Micra Transcatheter Pacing and LEADLESS II (Primary Results on Safety and Efficacy From the LEADLESS II-Phase 2 Worldwide Clinical Trial) trials have also demonstrated significant underutilization of LPMs in women at 41.2% and 38.2%, respectively [1, 2]. The post-approval Micra study also showed a lower utilization of LPMs in women as only 37.7% of such implantations occurred in them when compared to men [12]. Our current study of approximately 29,000 LPM implantations from real-world US practice showed persistent disparate utilization of LPMs based on sex as only 44.7% of such implantations occurred in women as compared to men. Unfortunately, our dataset is not equipped to analyze the etiologies behind the lower utilization of LPMs in women, but this should be investigated in future studies with a goal of narrowing such disparities.

Studies have shown women to be at a higher risk of complications after cardiac implantable electronic device implantations (CIEDs) as compared to men. In a Danish cohort of 5918 consecutive patients implanted with conventional transvenous CIEDs, women were found to be at a higher risk of any complication from the procedure (adjusted risk ratio 1.3, 95% CI 1.1–1.6) [6]. Most of these complications were related to pneumothorax and pericardial effusion from cardiac perforation. Similarly, in another study, Nowak et al. also demonstrated significantly increased complication rate after conventional transvenous pacemakers in women as compared to men (OR 1.3, 95% CI 1.1–1.5) [5]. In the i-LEAPER (International Leadless Pacemaker Registry) study which enrolled more than 1100 patients undergoing LPM, there was a trend towards an increased rate of major complication in women as compared to men although this difference did not reach statistical significance (hazard ratio 2.03, 95% CI 0.70–5.84). In another study of 489 patients undergoing LPM implantation at a single institution, Huang et al. demonstrated nearly double the rate of cardiac perforation in women as compared to men (0.8% vs. 0.4%) [13], consistent with the findings of our study that also showed twice the magnitude of pericardial effusion in women as compared to men after such implantations.

In our study, the rate of pericardial effusion was higher in women as compared to men after LPM implantations (4.7% vs. 2.3%, *p* < 0.01). Our earlier study analyzing the risk factors associated with significant pericardial effusion (defined as pericardial effusion requiring intervention) also demonstrated an increased risk of such effusions in women after LPM implantations [14]. It is plausible that women have a smaller right ventricular cavity when compared to men and that predisposes towards apical placement of such devices, where myocardial tissue is especially thinner, thus enhancing the risk of perforation and pericardial effusion. Thus, the use of contrast, transesophageal echocardiogram and steep fluoroscopic angles to aid in septal placement of such devices may help mitigate the risk of pericardial effusion after LPM implantations in women.

## Limitations

Our study findings must be viewed with certain important limitations. First, the NIS uses ICD codes to identify disease and procedures, which could introduce errors; however, the NIS employs a stringent data quality program to minimize these inaccuracies [8]. Second, our analysis is limited to short-term outcomes since the NIS records only data from the initial hospital admission and censor data after discharge. Third, detailed information on the leadless pacemaker implantation process, such as the use of contrast, type of leadless pacemaker implanted, or the operator and institutional expertise, is not available. Fourth, the NIS does not give any information on the electrical performance of LPMs. Fifth, NIS does not inform on the clinical circumstances leading to the implantation of the LPM.

## Conclusion

Our nationwide analysis of 29,000 LPM implantations depicted significant sex-based disparities in their utilization and outcomes. Only 44.7% of women received such implantations and the risk of mortality, major complications, prolonged length of stay, and increased hospitalization costs were higher in women after men after LPM implantations. These results, if proven by large-randomized trials, can have important clinical implications.
